# Magnetic field induced quantum phases in a tensor network study of Kitaev magnets

**DOI:** 10.1038/s41467-020-15320-x

**Published:** 2020-04-02

**Authors:** Hyun-Yong Lee, Ryui Kaneko, Li Ern Chern, Tsuyoshi Okubo, Youhei Yamaji, Naoki Kawashima, Yong Baek Kim

**Affiliations:** 1 Institute for Solid State Physics, University of Tokyo, Kashiwa, Chiba 277-8581 Japan; 2 Department of Display and Semiconductor Physics, Korea University, Sejong, 339-700 Republic of Korea; 3 Department of Physics, University of Toronto, Toronto, ON M5S 1A7 Canada; 4 Department of Physics, University of Tokyo, Tokyo, 113-0033 Japan; 5 Department of Applied Physics, University of Tokyo, Tokyo, 113-8656 Japan; 6 Perimeter Institute for Theoretical Physics, Waterloo, ON N2L 2Y5 Canada

**Keywords:** Magnetic properties and materials, Phase transitions and critical phenomena

## Abstract

Recent discovery of the half quantized thermal Hall conductivity in $\alpha$-RuCl${}_{3}$, a candidate material for the Kitaev spin liquid, suggests the presence of a highly entangled quantum state in external magnetic fields. This field induced phase appears between the low field zig-zag magnetic order and the high field polarized state. Motivated by this experiment, we study possible field induced quantum phases in theoretical models of the Kitaev magnets, using the two dimensional tensor network approach or infinite tensor product states. We find various quantum ground states in addition to the chiral Kitaev spin liquid occupying a small area in the phase diagram. They form a band of emergent quantum phases in an intermediate window of external magnetic fields, somewhat reminiscent of the experiment. We discuss the implications of these results in view of the experiment and previous theoretical studies.

## Abstract (web summary)

$\alpha$-RuCl${}_{3}$ is a candidate Kitaev spin liquid but whether or not this is realised depends on the details of the magnetic interactions. Here, the authors use state-of-the-art numerical methods to predict that for a large parameter region the Kitaev phase is supplanted by nematic paramagnetic phases.

## Introduction

Finding an unambiguous experimental evidence for quantum spin liquid has been a great challenge in the study of topological phases of matter^1,2^. Spin excitation spectra in quantum spin liquids, for example, consist of multiple excitations of underlying quasiparticles, namely, spinons. Hence, such spectra form a continuum and have no sharp excitations, which pose an inherent difficulty in identifying quantum spin liquids. In this context, the recent observation of half-quantized thermal Hall conductivity in the material $\alpha$-RuCl${}_{3}$ in an external magnetic field is a remarkable discovery^3^. $\alpha$-RuCl${}_{3}$ is a promising candidate for the gapless Kitaev spin liquid (KSL)^2,4–24^, which is the ground state of an exactly solvable spin model^25^. In the presence of magnetic field, it becomes the gapped chiral KSL, which supports the chiral Majorana edge mode^25^. The half-quantized thermal Hall conductivity can be regarded as a unique signature of this Majorana edge state.

Without magnetic field, however, $\alpha$-RuCl${}_{3}$ develops the zig-zag (ZZ) magnetic order at low temperatures^7–11^. Clearly, this must be due to the presence of spin interactions beyond the exactly solvable Kitaev model. A number of theoretical models are proposed and some minimal choices are the $K$-$\Gamma$-$\Gamma ^{\prime}$ and $K$-$\Gamma$-${J}_{3}$ models. Here $K$ is the ferromagnetic (FM) Kitaev interaction, and $\Gamma$ is the bond-dependent anisotropic interaction^10^. It is shown that a substantial $\Gamma$ is necessary to explain the large anisotropy of the magnetic susceptibility seen in experiments. The ZZ order arises due to another anisotropic interaction $\Gamma ^{\prime}$, which is induced by the trigonal distortion of Cl octahedra, or the third neighbor antiferromagnetic Heisenberg interaction ${J}_{3}$^26^. Hence, the central question is how the ZZ order would give away to the chiral KSL in the presence of magnetic field and whether this happens in these minimal models.

Previous results on the $K$-$\Gamma$-$\Gamma ^{\prime}$ model^27^ obtained from exact diagonalization (ED) on the 24-site cluster (when the magnetic field is tilted away from $[111]$ direction so that ${C}_{3}$ rotation symmetry is explicitly broken) and density matrix renormalization group (DMRG) on the 2-leg ladder geometry suggest that the chiral KSL is stabilized in a large window of magnetic field and $\Gamma /K$ between the ZZ and polarized phases. Another recent theoretical work on the classical model^28^, however, shows that there exist a multitude of complex magnetic orders with large unit cells in a similar window of intermediate magnetic fields. Many of these phases cannot be accommodated in small systems used in the ED and DMRG calculations mentioned above. In order to resolve this issue, theoretical studies of the quantum model in the thermodynamic limit are necessary.

In this article, we present the results of the infinite tensor product state (iTPS) studies on the $K$-$\Gamma$-$\Gamma ^{\prime}$ and $K$-$\Gamma$ models, which directly deal with the two-dimensional thermodynamic limit. Here we can treat the KSL and the classical complex magnetically ordered states on equal footing. Our study shows that the KSL only occupies a small corner in the magnetic field phase diagram. On the other hand, we find novel quantum phases, namely, the nematic paramagnets, to be emergent in an intermediate window of magnetic fields. Apart from providing a definite prediction for $\alpha$-RuCl${}_{3}$, our result also addresses the general question as to what are the possible quantum phases around the KSL in other spin–orbital entangled honeycomb magnets, which are described by similar theoretical models containing substantial $K$ and $\Gamma$ interactions. Below, we explain the phase diagram and discuss the nature of magnetic field-induced quantum phases.

## Results

### Model

We begin with the Hamiltonian of the $K$-$\Gamma$-$\Gamma ^{\prime}$ model^27–29^: $\hat{H}={\sum }_{{\langle ij\rangle }_{\gamma }}{\hat{H}}_{ij}^{\gamma }$ with

1 $${\hat{H}}_{ij}^{\gamma }=	\, -\frac{{\bf{h}}}{3}\cdot ({{\bf{S}}}_{i}+{{\bf{S}}}_{j})+K{S}_{i}^{\gamma }{S}_{j}^{\gamma }+\Gamma ({S}_{i}^{\mu }{S}_{j}^{\nu }+{S}_{i}^{\nu }{S}_{j}^{\mu })\\ 	\, +\Gamma ^{\prime} ({S}_{i}^{\mu }{S}_{j}^{\gamma }+{S}_{i}^{\gamma }{S}_{j}^{\mu }+{S}_{i}^{\nu }{S}_{j}^{\gamma }+{S}_{i}^{\nu }{S}_{j}^{\gamma }),$$

where ${\langle ij\rangle }_{\gamma }$ denotes the pair of the nearest neighbor sites, $i$ and $j$, on the $\gamma$-bond with $\gamma =x,y,z$ as depicted in the “Methods” section. The $K$ term is the isotropic Kitaev interaction. Here $(\gamma,\mu,\nu )$ forms a cyclic permutation of $(x,y,z)$ such that off-diagonal spin exchanges are represented by $\Gamma$ and $\Gamma ^{\prime}$ interactions. In both classical^28^ and quantum^27^ limits, a small $\Gamma ^{\prime}$ interaction induces the ZZ magnetic order at small magnetic fields, which gives away to other competing phases at larger magnetic fields. Throughout this article, we fix $\Gamma ^{\prime} =-0.03$ in units of $\sqrt{{K}^{2}+{\Gamma }^{2}}=1$ and focus on the FM Kitaev and antiferromagnetic $\Gamma$ interactions, i.e., $K\,<\,0$ and $\Gamma\, > \,0$, which is relevant to the material $\alpha$-RuCl${}_{3}$. The magnetic field is applied along the $[111]$ direction, i.e., ${\bf{h}}=h(1,1,1)/\sqrt{3}$. We also consider the effect of tilting the magnetic field from the $[111]$ direction. The Hamiltonian is invariant under the transformation ${C}_{6}{U}_{{C}_{6}}$: $[{C}_{6}{U}_{{C}_{6}},H]=0$, where ${C}_{6}$ denotes $6{0}^{\circ }$ lattice rotation about the center of the plaquette while ${U}_{{C}_{6}}$ cyclically permutes the components of the spin operator, i.e., ${S}^{x}\to {S}^{y}\to {S}^{z}\to {S}^{x}$. For simplicity, we refer to this symmetry as the rotational symmetry.

### Identification of each phase

**Fig. 1:**
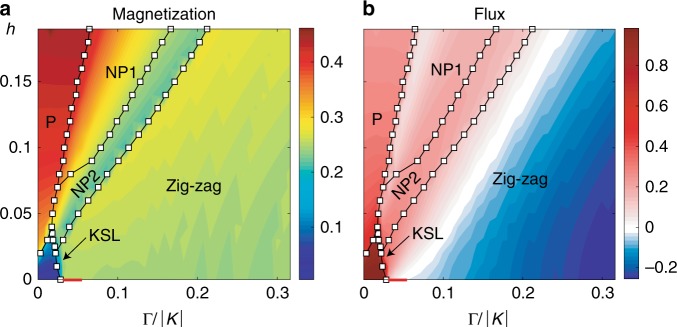
Ground-state phase diagram. **a** The magnetization and **b** the flux expectation value of the $K$-$\Gamma$-$\Gamma ^{\prime}$ model. Here KSL stands for the chiral Kitaev spin liquid, P for a spin-polarized phase, and NPs for nematic paramagnet phase. The red solid line at $h=0$ denotes a ferromagnetic phase (see text for details).

To determine the phase boundaries and characterize each phase, we optimize the iTPS using the imaginary time evolution (ITE)^30^ and measure the energy density $E=\langle H\rangle /{N}_{s}$, magnetization $M\equiv {N}_{s}^{-1}{\sum }_{i}^{{N}_{s}}\sqrt{{\langle {{\bf{S}}}_{i}\rangle }^{2}}$, and flux $W\equiv {N}_{p}^{-1}{\sum }_{p}^{{N}_{p}}\langle {\hat{W}}_{p}\rangle$. Here ${\hat{W}}_{p}={\hat{\sigma }}_{1}^{x}{\hat{\sigma }}_{2}^{y}{\hat{\sigma }}_{3}^{z}{\hat{\sigma }}_{4}^{x}{\hat{\sigma }}_{5}^{y}{\hat{\sigma }}_{6}^{z}$ is the flux operator^25^ on a plaquette $p$, the site indices $1-6$ are defined in the “Methods” section, and ${N}_{s(p)}$ is the number of sites (plaquettes) in the system. As shown in the phase diagram Fig. 1, we identify five distinct phases, i.e., KSL, polarized (P), nematic paramagnetic (NP1 and NP2), and ZZ phases in the parameter region $0\, <\, \Gamma /| K| \le 0.3$ and $0<h\le 0.2$.

### Small extent of KSL in field

**Fig. 2:**
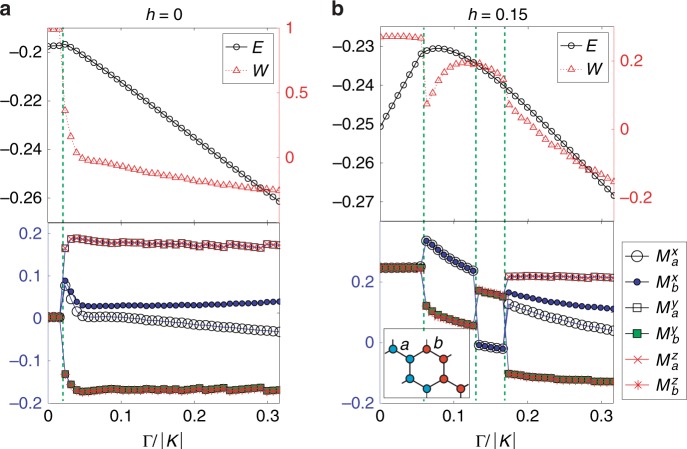
Distinguishing phases. Plots of variational energy $E$, flux $W$ (upper), and components of magnetization ${M}_{a,b}^{\gamma }$ (lower) at **a** $h=0$ and **b** $h=0.15$ as a function of $\Gamma /| K|$ with $\Gamma ^{\prime} =-0.03$. The green dotted lines specify the phase boundaries. The information on numerical parameters is given in the “Methods” section.

First, the KSL ground state survives only in a small corner of the phase diagram. In the KSL phase, the magnetization and the fluctuation of vortices are suppressed, i.e., $M\ll 1/2$ and $W\approx 1$ as shown in Figs. 1 and 2a. It disagrees with the largely extended KSL phase observed in the 24-site ED and DMRG studies on the 2-leg ladder system in ref. ^27^. The discrepancy may imply that taking the thermodynamic limit is important. At zero field, there is a transition from KSL to a FM phase where spins are aligned in the $[1\bar{1}\bar{1}]$ direction (red solid line in Fig. 1). However, with a very weak magnetic field ($h=0.005$), the FM phase disappears, and a direct phase transition from KSL to ZZ occurs. With increasing $h$, the transition from KSL to the P phase occurs at a finite $h$, where spins start aligning in the $[111]$ direction. The fate of the FM states will be discussed in detail later. We have found that the field-induced phase transition with $(\Gamma,\Gamma ^{\prime} )=(0,-0.03)$ occurs at ${h}_{c}^{K\Gamma ^{\prime} }\approx 0.011$, which is smaller than ${h}_{c}^{K}\approx 0.02$^31–35^ of the pure Kitaev model (see Supplementary Note 5).

### Nematic paramagnetic phases

As $\Gamma$ increases, the magnetic field-induced phase is no longer the KSL. The ZZ order gives away to interesting intermediate phases NP1 and NP2  (Fig. 1) before the system enters the P phase at high field. Both phases are nematic in the sense that the rotational symmetry is spontaneously broken down to the ${C}_{2}$ rotational symmetry. More specifically, the local energy ${E}^{\gamma }=\langle {\hat{H}}_{ij}^{\gamma }\rangle$ depends on the direction of bond $\gamma$: ${E}^{x}\,<\,{E}^{y}={E}^{z}$ in NP1 while ${E}^{x}\,> \,{E}^{y}={E}^{z}$ in NP2. It also leads to the anisotropic magnetization, i.e., ${M}^{x}\, \ne \,{M}^{y}={M}^{z}$ etc., as presented in Fig. 2b.

In the classical limit, the 8-, 18-, and 32-site magnetic orders are stabilized in a similar parameter regime^28^. Our result indicates that strong quantum fluctuation melts the competing large unit-cell orders, leading to the restoration of the translational symmetry, while the rotational symmetry remains broken. We have also found that the NP phases appear and survive down to almost zero field limit in the $K$-$\Gamma$ model as shown below. By increasing the accuracy of the iTPS representation (see Supplementary Note 7), we have confirmed that the NP states are quantum paramagnet and develop finite magnetization only in the presence of the field.

**Fig. 3:**
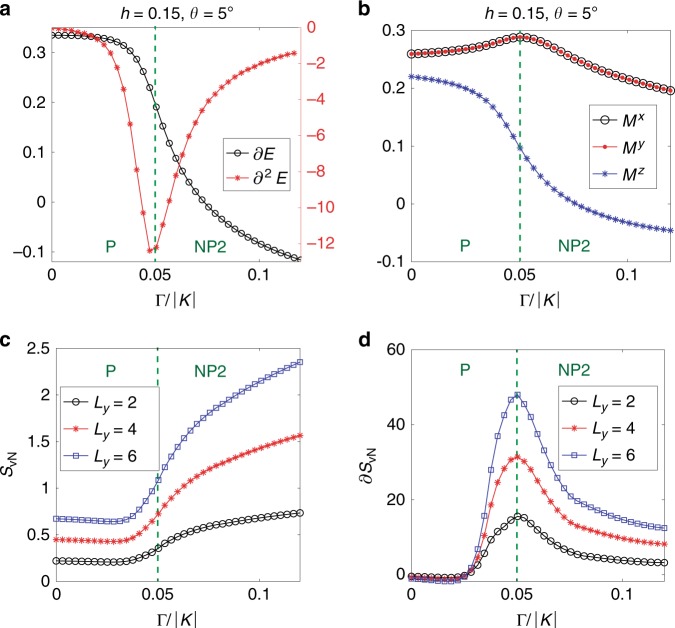
Transition between the P and NP2 phases. Plots of **a** the first and second derivatives of the energy density, **b** magnetization components, **c** entanglement entropy (${S}_{{\rm{vN}}}$), and **d** its first derivative with the tilting angle $\theta ={5}^{\circ }$ and $h=0.15$. Here ${S}_{{\rm{vN}}}$ is measured on the cylinder geometry with the circumference ${L}_{y}$. The information on numerical parameters is given in the “Methods” section.

In the [111] magnetic field, the nature of the transition between P and NP1 phases is not clear. Even though the local observables show finite jumps at the transition, these are not very distinctive compared to other transitions and may originate from the inherently biased optimization in ITE, which is analyzed carefully in Supplementary Note 2. The non-triviality of the NP phases is revealed by tilting the magnetic field slightly toward the $[11\bar{2}]$ direction. Figure 3a presents the optimized energy and its second derivative with respect to $\Gamma /| K|$ at the tilting angle $\theta ={5}^{\circ }$. Notice that, owing to the tilted field, the model breaks the rotational symmetry explicitly, and thus there is no remaining symmetry discriminating the P and NP phases. Nevertheless, the second derivative of the energy strongly suggests a continuous phase transition between the P and NP2 phases (see Fig. 3b) at $\Gamma /| K| \approx 0.05$. Note that the tilted field with $\theta \,> \,0$ leads to a transition from the P phase directly to the NP2 phase. On the other hand, tilting the field in the opposite direction ($\theta\, <\,0$) favors the NP1 phase and therefore gives rise to a transition from the P phase to the NP1 phase (see Supplementary Note 6). The continuous nature of these transitions can be seen even more clearly in the entanglement entropy (EE)^36–38^. The boundary theory of TPS^39^ has been employed to measure the EE on the cylinder geometry with the circumference ${L}_{y}$, and the result is presented in Fig. 3c, d. The NP1 state is highly entangled and its EE increases with $\Gamma$, while the P state has a low and constant EE. The first derivative of the EE exhibits a peak at the same point as that of the second derivative of the energy, and it becomes sharper with increasing ${L}_{y}$ and the accuracy of the variational ansatz (see Supplementary Note 3). Therefore, we conclude that there is a continuous transition between the P and NP2 phases at $\Gamma /| K| \approx 0.05$. As mentioned above, the P and NP phases cannot be distinguished by conventional symmetries, thus the continuous transition implies a topological phase transition from the trivial phase (P) to a topological or non-trivial phase (NP2). It is worth noting that the tilted field makes the numerical optimization much more stable as analyzed in Supplementary Fig. 2.

### *K*-${\mathbf{\Gamma}}$ model

**Fig. 4:**
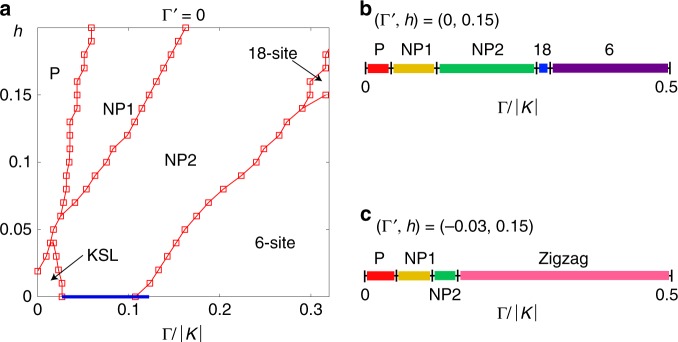
Comparison to the phase diagram of the *K*$-\, {\mathbf{\Gamma}}$ model. **a** Phase diagram at $\Gamma ^{\prime} =0$ as functions of $\Gamma /| K|$ and $h$. NP1 and NP2 phases are larger than those at $\Gamma ^{\prime} =-0.03$ since the zig-zag phase is less dominant. Complexed magnetic phases appear when $\Gamma /| K| \,\gtrsim \,0.3$ and $h\,\gtrsim \,0.15$. The blue solid line at $h=0$ denotes a ferromagnetic phase (see text for details). Larger $\Gamma$ phase diagrams at **b** ($\Gamma ^{\prime},h$) $=(0,0.15)$ and **c** ($\Gamma ^{\prime},h$) $=(-0.03,0.15)$. The information on numerical parameters is given in the “Methods” section.

Finite $\Gamma ^{\prime}$ is responsible in stabilizing the ZZ order at low fields. When $\Gamma ^{\prime} =0$, we may expect a more significant competition between various phases, including the complex classical magnetic orders. We find that the NP phases are already present in the $K$-$\Gamma$ model as shown in the phase diagram in Fig. 4. On the other hand, the complex magnetic orders with large magnetic unit cells appear for sufficiently large $\Gamma$ (typically $\Gamma /| K|\, \gtrsim \,0.3$). For example, the 6-site order phase appears at lower field $h\,\lesssim \,0.15$ while the 18-site order phase appears at higher field $h\,\gtrsim \,0.15$ as presented in Fig. 4a, b. These are the same magnetic orders reported in the classical phase diagram^28^. Quantum fluctuations seem to favor NP1 and NP2 phases at small $\Gamma$ and push the classical orders to the parameter region with larger $\Gamma$. As in the case of $\Gamma ^{\prime} =-0.03$, the FM phase appears between the KSL and the 6-site order at $h=0$. This reminds us of a FM phase in a tiny area of the phase diagram in the variational Monte Carlo study in ref. ^24^. However, the NP2 state is almost degenerate with FM phase in this region, i.e., the energy difference is only $\Delta E \sim O(1{0}^{-4})$. Moreover, even this tiny energy difference is decreasing as the accuracy of the iTPS further increases (see Supplementary Note 8). With these results and given that the FM quickly loses to NP2 with a very small $h$, NP2 may become a stable ground state or degenerate with the FM phase at $h=0$ as the variational iTPS approaches to the exact ground state. The NP phases are reminiscent of the $K\Gamma$ spin liquid reported in the previous iDMRG study^20^, where the rotational symmetry is broken in similar manner.

## Conclusion

We have used iTPS optimization to investigate the field-induced quantum phases in the $K$-$\Gamma$-$\Gamma ^{\prime}$ model. Apart from the well-established chiral KSL, we discover the stabilization of the nematic paramagnets NP1 and NP2 at intermediate magnetic fields. The NP phases break lattice rotational symmetry spontaneously and take place between the low-field ZZ magnetic order and the high-field polarized state. In contrast to the previous 24-site ED and 2-leg ladder DMRG study^27^, the KSL is found to survive only in a small corner of the phase diagram. Instead, the NP phases occupy a large portion of the phase diagram and hence are more likely to be observed. We propose that, to probe the nematic paramagnets experimentally, one could measure longitudinal thermal conductivity and magnetic susceptibility over the in-plane directions, which would display the breaking of ${C}_{3}$ symmetry. We also find that the NP phases are already present in the $K$-$\Gamma$ model in zero and finite magnetic field. The NP phases in the $K$-$\Gamma$ model give away to the complex magnetic orders with large unit cells when $\Gamma /| K|$ becomes large, making contact with the classical phase diagram reported earlier.

In order to clarify the nature of the NP phases, we examine the effect of tilting the magnetic field ($\theta ={5}^{\circ }$ from the [111] direction). Here the transition between the polarized (P) and NP2 phases is continuous, judging from the singular behaviors in the second derivative of the energy and the first derivative of the EE. Since ${C}_{3}$ is broken in both of the P and NP2 phases in the tilted field, the continuous transition would imply that NP2 is not a trivial product state. This leaves the interesting possibility that the NP phases are non-trivial topological states. The precise nature and thermal Hall response of these states would be interesting subjects of future study.

## Methods

### iTPS and ITE

**Fig. 5:**
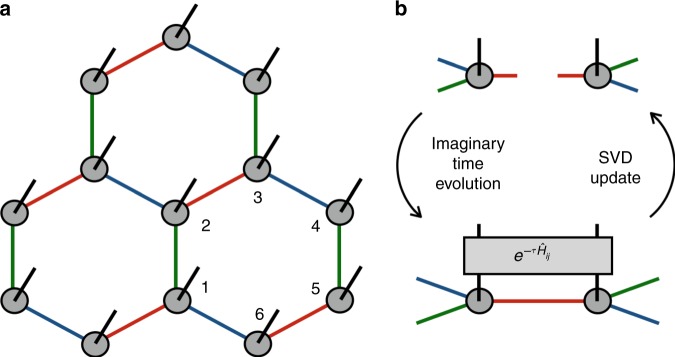
Methods. Schematic figures of **a** the honeycomb TPS where the $x$-, $y$- and $z$-bonds defined in Eq. (1) are specified by red, blue and green colors, respectively, and **b** the optimization and update processes of the local tensor, where the black solid line denotes the physical degrees of freedom and ${e}^{-\tau {\hat{H}}_{ij}}$ is the local imaginary time evolution operator (see text for detail).

In order to carve out the ground-state phase diagram, we employ the iTPS representation^40^ on the honeycomb lattice and optimize it with respect to the Hamiltonian in Eq. (1). The iTPS wavefunction ${\psi }_{\{{s}_{i}\}}={\rm{tTr}}{\prod }_{i}{[{T}_{i}]}_{{\alpha }_{i}{\beta }_{i}{\gamma }_{i}}^{{s}_{i}}$ is illustrated in Fig. 5a, where ${s}_{i}$ denotes the spin state at site $i$, and ${\rm{tTr}}$ represents the trace over the virtual indices $({\alpha }_{i},{\beta }_{i},{\gamma }_{i})$ of the local tensor ${T}_{i}$. The accuracy of the iTPS representation becomes better as the dimension of the virtual indices, or the bond dimension $D$, increases. The ITE is adopted for optimization, i.e., the two-site gate ${e}^{-\tau {\hat{H}}_{ij}^{\gamma }}$ is applied on every bond with fixed $\tau =0.01$. Then the local tensors are updated by the singular value decomposition^30^. Iterating this two-step procedure (Fig. 5b) drives the initial state into the ground state.

Since the ITE with such a simple update can be easily biased by the initial choice of ${T}_{i}$, we optimize various trial states and choose the lowest energy state as the ground state. We consider the string gas (SG) represetation of the KSL in ref. ^41^ and the classical magnetic orders found in ref. ^28^. Note that the ITE starting from the SG state, i.e., $\left|\psi \right\rangle \approx {({e}^{-\tau \hat{H}})}^{{\mathcal{N}}}\left|{\rm{SG}}\right\rangle$, provides the lowest energy states near the pure Kitaev limit, which allows us to determine the KSL phase. We also include the FM [111] state (FM[111]), where all spins are aligned in the $[111]$ direction, ZZ, and 6- and 18-site magnetic orders found in ref. ^28^. In addition, we use the FM[100], FM[011], and FM[1$\bar{1}\bar{1}$] states as other possible initial states. Details of the initial states are provided in Supplementary Note 1. Owing to the complexity, we did not take into account the 32- and 50-site magnetic order discovered in the classical phase diagram^28^, which might be relevant for larger $\Gamma$ and $h$ than the parameter region considered in this work. To measure the physical quantities after the optimization, we employ the corner transfer matrix renormalization group (CTMRG) method^42–44^. The parallel C++ library mptensor^45^ is utilized to perform CTMRG and ITE. The main results in this article are obtained with the bond dimension $D=6$. It turns out that the phase diagram and physical quantities do not change much by increasing $D$ as discussed in Supplementary Information.

## Supplementary information

Supplementary Information

## Data Availability

All relevant data in this paper are available from the authors upon reasonable request.

## Code availability

All numerical codes in this paper are available from the authors upon reasonable request.
