# Supplementary material for: Magnetic field induced quantum phases in a tensor network study of Kitaev magnets
Source: Nat Commun. 2020 Apr 2;11:1639. doi: 10.1038/s41467-020-15320-x (PMC7118087; doi:10.1038/s41467-020-15320-x)
Supplement: Supplementary file 1 — Supplementary Information [file 41467_2020_15320_MOESM1_ESM.pdf]

Supplementary Information: Magnetic field induced quantum phases in a tensor network study of Kitaev magnets

Hyun-Yong Lee,[1,2] Ryui Kaneko,[1] Li Ern Chern,[3] Tsuyoshi Okubo,[4]
Youhei Yamaji,[5] Naoki Kawashima,[1] and Yong Baek Kim[3,6]

[1]*Institute for Solid State Physics, University of Tokyo, Kashiwa, Chiba 277-8581, Japan*
[2]*Department of Display and Semiconductor Physics, Korea University, Sejong 339-700, Republic of Korea*
[3]*Department of Physics, University of Toronto, Toronto, Ontario M5S 1A7, Canada*
[4]*Department of Physics, University of Tokyo, Tokyo 113-0033, Japan*
[5]*Department of Applied Physics, University of Tokyo, Tokyo 113-8656, Japan*
[6]*Perimeter Institute for Theoretical Physics, Waterloo, Ontario N2L 2Y5, Canada*

(Dated: February 21, 2020)

We define the unit cell structures and the magnetic moment on each sublattice of the initial magnetic states used in the optimization. Then, the dependence of the optimized states on the initial state and the bond dimension are discussed. In addition, we provide details of the field induced phase transition, the critical field strength h_c at $\Gamma = 0$ and -0.03, and tilting the magnetic field towards the [001]-direction ($\theta < 0$).

PACS numbers:

Supplementary Note 1: Unit cell structures of initial magnetic states

We present the details of the classical magnetic orders used as the initial states in the imaginary time evolution optimization. We have considered ten different initial states, which are the string gas, FM[111], FM[100], FM[011], zigzag, 6-site, 8-site, 18-site1, 18-site2 and 18-site3 states. The definition of the string gas state is given in Supplementary Ref. 1. The FM[abc] state denotes the classical product state where all spins are aligned in the [abc]-direction. The remaining states are classical product states defined on larger unit cells. The structure of the magnetic unit cells are depicted in Supplementary Fig. 1, and the (normalized) magnetization components are given below.

- zigzag
  1:$(0, 1/\sqrt{2}, 1/\sqrt{2})$, 2:$(0, -1/\sqrt{2}, -1/\sqrt{2})$
- 6-site
  1:$(0, 1/\sqrt{2}, 1/\sqrt{2})$, 2:$(1/\sqrt{2}, -1/2, -1/2)$, 2:$(-1/\sqrt{2}, -1/2, -1/2)$
- 8-site
  1:$(1/\sqrt{2}, 1/\sqrt{2}, 0)$, 2:$(-1/\sqrt{2}, 1/\sqrt{2}, 0)$, 3:$(1/\sqrt{2}, 1/\sqrt{2}, 0)$, 4:$(-1/\sqrt{2}, -1/\sqrt{2}, 0)$
- 18-site1
  1: $(0.694426963314, 0.508808015180, 0.508808015180)$, 2: $(0.917670696610, 0.280963069267, 0.280963069267)$,
  3: $(0.360919338886, 0.360919338886, -0.859927009482)$, 4: $(0.280963069267, 0.917670696610, 0.280963069267)$,
  5: $(0.508808015180, 0.694426963314, 0.508808015180)$, 6: $(0.508808015180, 0.508808015180, 0.694426963314)$,
  7: $(0.360919338886, -0.859927009482, 0.360919338886)$, 8: $(0.280963069267, 0.280963069267, 0.917670696610)$
- 18-site2
  1: $(0.358086657424, 0.627886711621, 0.691037063510)$, 2: $(0.317167993518, 0.662906654162, 0.678202942898)$,
  3: $(0.058719049073, 0.953736826431, 0.294852741526)$, 4: $(0.076804072888, 0.231146393012, 0.969882714242)$,
  5: $(0.830779222896, 0.194767830929, 0.521412864090)$, 6: $(0.691037063510, 0.627886711621, 0.358086657424)$,
  7: $(0.478273748206, 0.828840613223, 0.290305803678)$, 8: $(0.521412864090, 0.194767830929, 0.830779222896)$,
  9: $(-0.969882714242, 0.231146393012, 0.0768040728)$, 10: $(-0.917656299286, 0.261691990911, 0.2990388240)$,
  11: $(0.263587705748, 0.179270501689, 0.947831002132)$, 12: $(0.290305803678, 0.828840613223, 0.478273748206)$,
  13: $(0.723791516161, 0.349229309838, 0.595117408823)$, 14: $(0.947831002132, 0.179270501689, 0.263587705748)$,
  15: $(0.299038824024, 0.261691990911, 0.917656299286)$, 16: $(0.294852741526, 0.953736826431, 0.058719049073)$,
  17: $(0.678202942898, 0.662906654162, 0.317167993518)$, 18: $(0.595117408823, 0.349229309838, 0.723791516161)$
- 18-site3
  1: $(0.256377480005, 0.705963966202, 0.660216226831)$, 2: $(0.877902927220, 0.452899788357, 0.155461352380)$,
  3: $(0.639181668028, 0.427660602011, 0.639181668028)$, 4: $(0.660216226831, 0.705963966202, 0.256377480005)$,

5: (0.691497143275, 0.208957894528, 0.691497143275), 6: (0.693921708246, 0.192211668868, 0.693921708246),
7: (0.516130572975, 0.834166479598, 0.194359244588), 8: (0.194359244588, 0.834166479598, 0.516130572975),
9: (0.155461352380, 0.452899788358, 0.877902927220)

Supplementary Note 2: Initial state dependence of imaginary time evolution

As mentioned in the main text, the imaginary time evolution method combined with the simple local update of tensors is easily biased by the choice of the initial state. Here, we discuss the initial state dependence of the optimized states. First, we find that the ground states in the chiral Kitaev spin liquid phase are obtained only from the string gas (SG) initial state[1], as shown in the left panel of Supplementary Fig. 2. As one can see, the optimized states starting from the SG state has significantly lower energy than others, thus the choice of the initial state is very important in the KSL phase. Other magnetic initial states do not evolve to the correct ground states. We should also notice that the correct phase boundary and its nature might be concealed by such a biased optimization.

The center panel of Supplementary Fig. 2 presents the magnetizations of the optimized states resulting from the initial FM[111] and FM[100] states, which provide the ground states in the polzarized (P) and NP phases, respectively. In the blue (red) shaded region, both initial states converge to (almost) identical states such that the magnetizations agree. However, the initial FM[111] state favors the nature of the P state above the phase boundary, whereas the FM[100] state favors the nature of the NP1 state below the phase boundary. Because of these hysteresis-like behavior of the optimization, the phase transition always appears to be first order, since the ground state is identified through energy comparison. Nonetheless, we find that tilting the magnetic field leads to an unambiguous optimization which does not depend on the initial state around the phase boundary between the P and NP phases, as shown in the right panel of Supplementary Fig. 2. Based on the well converged results, we conclude that the phase transition between the P and NP phase in a tilted field is continuous, which strongly suggests the non-triviality of the NP phases. That is, the NP states may not be smoothly connected to a trivial product state.

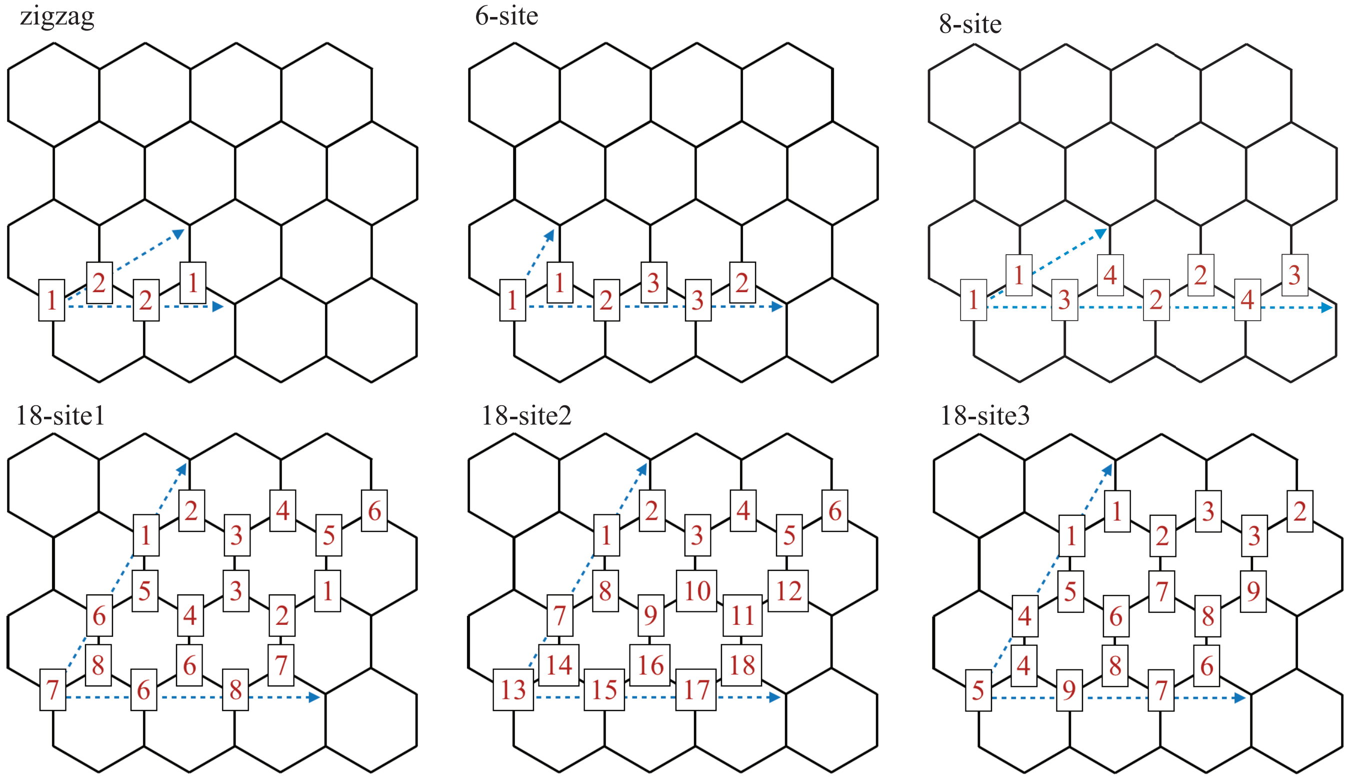

Supplementary FIG. 1: The unit cell structures of various magnetic states, where the sublattices are labeled with numbers, and the blue arrows denote the primitive vectors.

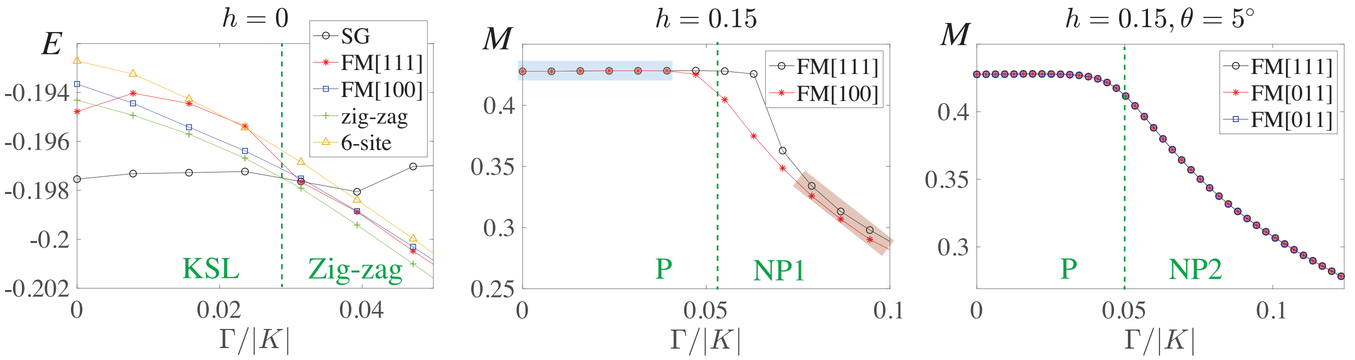

Supplementary FIG. 2: (left) The variational energies depending on various initial states at $(\Gamma', h) = (-0.03, 0)$, the magnetizations of the optimized states obtained from (center) the initial FM[111], FM[100] states with $\theta = 0$ and (right) the initial FM[111], FM[011], FM[100] states with $\theta = 5°$. The green dotted line indicates the phase boundary.

Supplementary Note 3: Bond dimension dependence of Nematic Paramagnet phases

In this section, we discuss the bond dependence of the nematic paramagnet (NP) phases. In most cases, the NP1 phase is obtained by optimizing the initial FM[100] state, where all spins are aligned in the [100]-direction, while the initial FM[011] state leads to the NP2 phase. Supplementary Fig. 6 presents the variational energy $E = \langle H \rangle / N$, the size of the magnetization $M = \sqrt{(M^x)^2 + (M^y)^2 + (M^z)^2}$ and the magnetization components $M^\gamma = (1/N) \sum_i \langle S_i^\gamma \rangle$ for each of the initial FM[100] and FM[011] states with bond dimensions $D = 4$, 6 and 8. The NP1 phase does not depend strongly on the bond dimension, that is, the spin configurations are already captured well by the $D = 4$ ansatz as one can see in the upper right panel of Supplementary Fig. 6. The $D = 6$ and $D = 8$ ansatze give almost identical energies and magnetizations. On the other hand, the NP2 phase seems to require larger bond dimensions to converge. Notice that the $D = 4$ ansatz does not represent the NP2 states well as one can see in the magnetization components (right panels of Supplementary Fig. 6). Furthermore, the $D = 6$ and $D = 8$ states show some discrepancies in the size of magnetization and z-component of the magnetization, though their variational energies are quite close each other. We also have found that the variational energies of the zigzag and polarized phases do not change much for $D \geq 6$. Therefore, we believe that the phase diagram Fig. 2 in the main text will be more or less the same should larger bond dimensions are used.

We have also checked the bond dependence of the second derivative of variational energy and magnetization at $(\Gamma', h, \theta) = (-0.03, 0.15, 5°)$, where θ denotes the tilting angle of the magnetic field (see the main text). The results are shown in Supplementary Fig. 3 (a) and (b), and it is clear that the peaks become sharper at larger bond dimension ($D = 6$). The peaks in the first derivative of the entanglement entropy also becomes sharper with larger bond dimension, as shown in Supplementary Fig. 3 (d).

Supplementary Note 4: Larger unit-cell magnetic states

As mentioned in the main text, the larger unit cell magnetic states, e.g., the 6-site, 8-site and 18-site orders found in Supplementary Ref. 2, restore the translational symmetry and converge to the polarized state, NP states and even zigzag states after the optimization. On the other hand, they could also be stuck in some local minima, thus their variational energies are significantly higher than the ground state energy. Supplementary Fig. 4 shows the variational energies obtained from the initial 6-site, 8-site and 18-site magnetic states at $(\Gamma', h) = (-0.03, 0.1)$. As one can see in the left panel, the optimized states from the 18-site order are trapped in bad local minima such that the variational energies are far away from those of the ground states. We have performed the imaginary time evolution (ITE) up to $30,000$ steps with $\tau = 0.01$ (see main text). However, the variational energies do not approach the ground state energy throughout the ITE. On the other hand, the 6-site initial states converge to the P and NP states well during ITE by restoring the translational symmetry, i.e., the 2-site unit-cell is recovered. The center panel of Supplementary Fig. 4 shows that the $M^\gamma_{1,2,3}$, where the subscript denotes the sublattice defined in Supplementary Fig. 1, becomes independent on the sublattice, i.e., the translational symmetry is restored (even though it is not perfect). The spin configurations and energy also become similar to the NP1 states obtained from the FM[100] and FM[111] states. Similarly, the 8-site initial state becomes NP1-like states in $0.2 \lesssim \Gamma/|K| \lesssim 0.5$, while it becomes similar to the zigzag state if $\Gamma/|K| > 0.7$ as shown in the right panel of Supplementary Fig. 4 such that two zigzag unit cells are realized

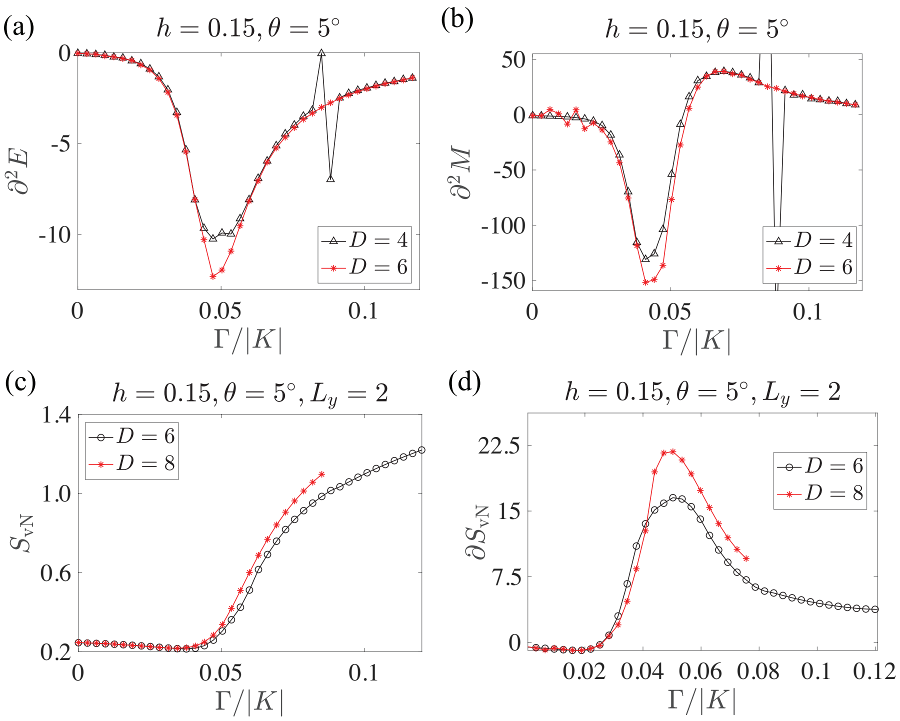

Supplementary FIG. 3: Bond dependence of (a) the second derivative of variational energy, (b) magnetization, (c) the entanglement entropy and (d) its second derivative at $(\Gamma', h, \theta) = (-0.03, 0.15, 5^\circ)$ with the tilted field $\theta = 5^\circ$ (see text). The entanglement entropy is measured on the cylinder geometry with the circumference $L_y = 2$.

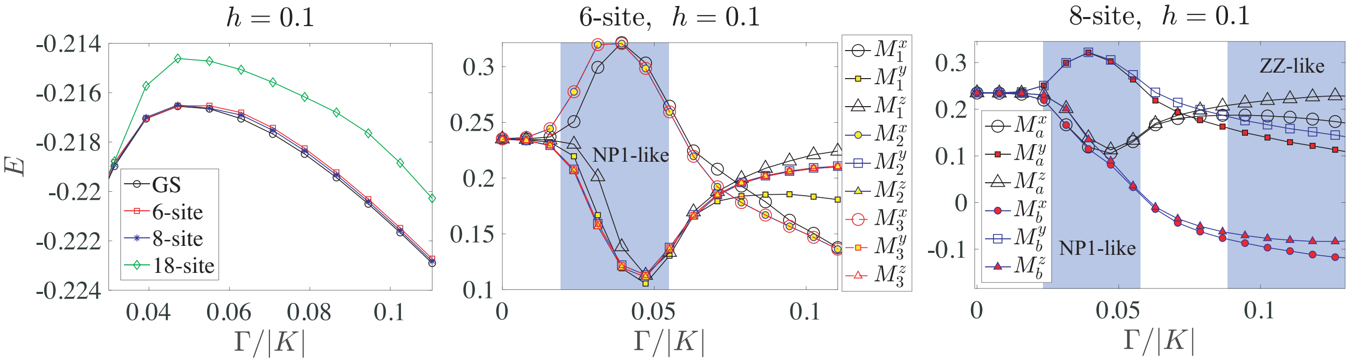

Supplementary FIG. 4: Plots of (left) the variational energies of the large unit-cell anstaze, i.e., the 6-site, 8-site and 18-site1 state, and the magnetization components on (center) three different sublattices of the 6-site order and (right) two different sublattices of the 8-site order.

in the eight sublattices.

In contrast, the zigzag state is no longer the ground state at $\Gamma' = 0$, and the larger unit-cell magnetic states appear at sufficiently large Γ. The 6-site and two 18-site orders are competitive, and the 6-site (18-site) order is favorable at lower (higher) magnetic fields. This tendency is consistent with the classical phase diagram[2].

Supplementary Note 5: Critical magnetic field at $\Gamma = 0$

In this section, we discuss the effect of the Γ' interaction on the critical field strength h_c, at which a phase transition occurs between the Kitaev spin liquid (KSL) and the polarized phase, with $\Gamma = 0$. Previous studies reported $h_c \approx 0.02$ from DMRG study[3] and $h_c \approx 0.025$ from 24-site ED studies[4,5] without the Γ' interaction. Using the string gas initial state, we have achieved a similar value $h_c \approx 0.01925$ at $\Gamma' = 0$, as shown in Supplementary Fig. 7. The transition seems to be first order at which the magnetization M and the flux expectation value W are discontinuous (see the

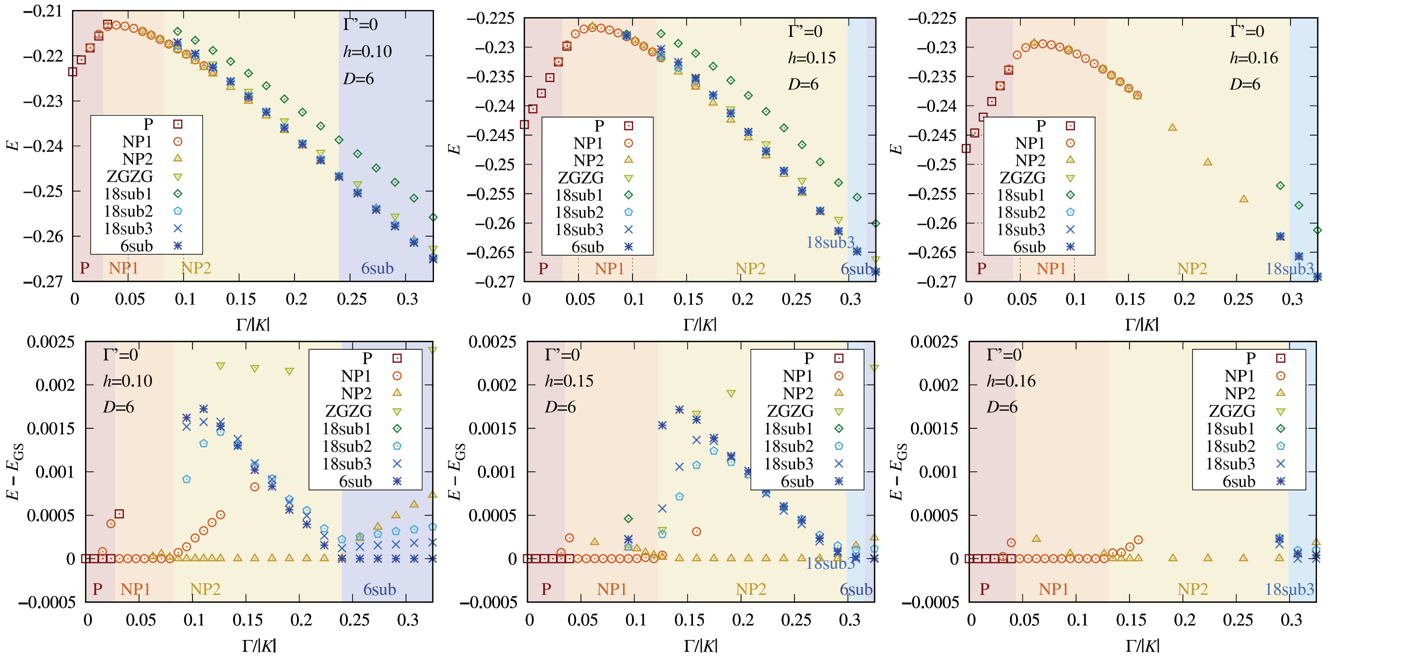

Supplementary FIG. 5: Comparison of (upper) energies and (lower) energy differences of ground-state candidates for (left) $h = 0.1$, (center) $h = 0.15$, and (right) $h = 0.16$ at $\Gamma' = 0$ and $D = 6$.

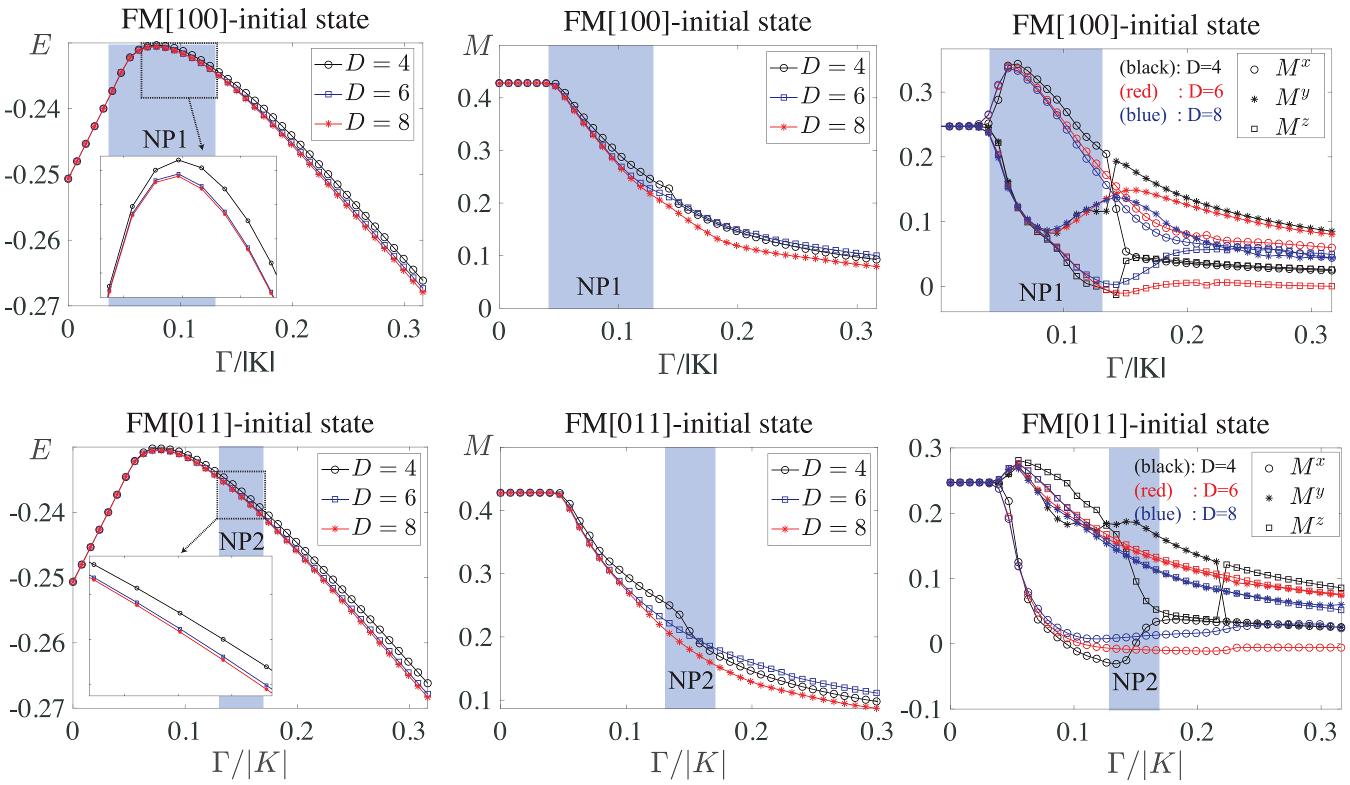

Supplementary FIG. 6: Bond dependence of (left) the variational energy $E = \langle H \rangle / N$, (center) the size of magnetization $M = \sqrt{(M^x)^2 + (M^y)^2 + (M^z)^2}$ and (right) the magnetization component $M^\gamma = (1/N) \sum_i \langle S_i^\gamma \rangle$ of the optimized states from the initial magnetic (upper) FM[100] and (lower) FM[011] states at $(\Gamma', h) = (-0.03, 0.15)$. Here, each optimized state becomes the ground state in the blue shaded region belonging to the NP1 and NP2 phases, respectively.

yellow dotted line). The presence of the Γ' interaction makes the KSL phase less stable, such that the field induced

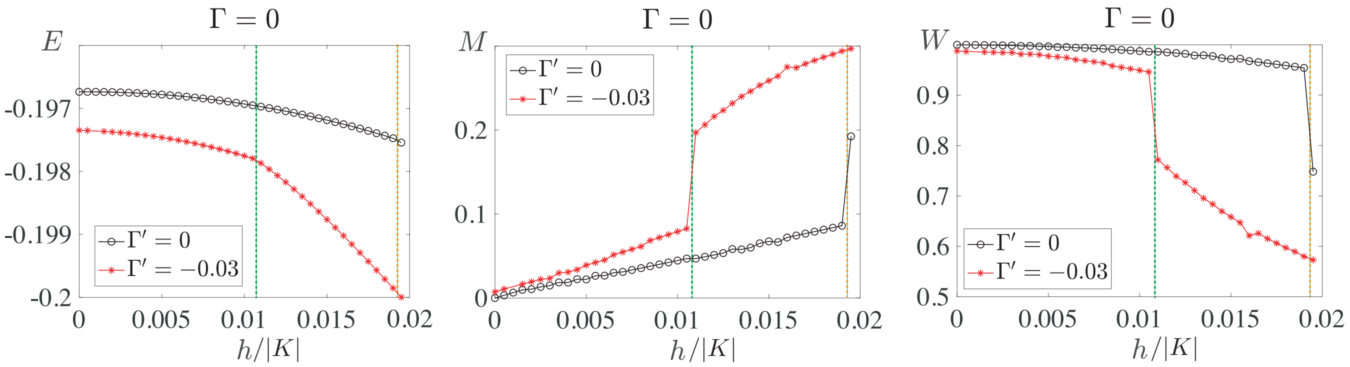

Supplementary FIG. 7: Plots of (left) the variational energy, (center) magnetization and (right) flux expectation value as a function of the field strength h at $\Gamma = 0$. The results are obtained from the string gas initial state with bond dimension $D = 6$. The yellow and green dotted lines stand for the critical field, at which the phase transition occurs between the Kitaev spin liquid and polarized phases, with $\Gamma' = 0$ and $\Gamma' = -0.03$, respectively.

transition occurs at lower h, i.e., $h_c(\Gamma' = -0.03) \approx 0.01075$. See the green dotted line in Supplementary Fig. 7.

Supplementary Note 6: Tilting field toward to the [001]-direction

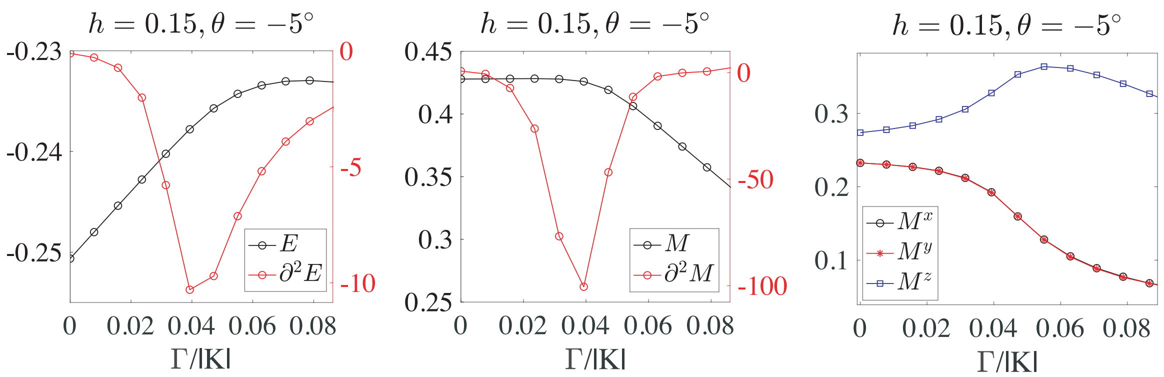

Supplementary FIG. 8: Plots of (left) the variational energy and its second derivative, (center) magnetization and its second derivative, and (right) magnetization components as a function of the field strength $\Gamma/|K|$ at $(\Gamma', h, \theta) = (-0.03, 0.15, -5°)$. The results are obtained from the $D = 6$ ansatze.

In the main text, we consider tilting the field towards the $[11\bar{2}]$-direction (θ: tilting angle), which leads to the phase transition between the polarized and NP2 phases without going through the NP1 phase, as shown in Fig. 4. Here, we present the results of tilting the field towards the [001]-direction, i.e., $\theta = -5°$, in Supplementary Fig. 8. It leads to the continuous-like phase transition between the polarized and NP1 phases as one expect.

Supplementary Note 7: Magnetization of the nematic paramagnet phase

In the K-Γ-Γ' model, the NP phases appear at finite magnetic fields and are not stabilized in the zero field limit. Without the Γ' interaction, the NP phases become wider and survive down to almost zero field. Here, we show that the NP2 state becomes non-magnetic at zero field. Even though the NP states seem to have spontaneous magnetizations, it is an artifact of the finite bond dimension D. Supplementary Fig. 9 presents the D-scaling of the variational energy and magnetization at $\Gamma/|K| = 0.03$ of the K-Γ model. As one can see, even with $D \geq 10$ which is a considerably large bond dimension, the energy and the magnetization exhibit a visible evolution instead of a convergent behavior. To extrapolate the magnetization at $D \to \infty$, we have fitted the magnetization at $D = 6, 8, 10$ and 12 with a linear function (see the red solid line in the center panel). It strongly indicates a zero magnetization at $D \to \infty$, and thus the NP phases only develop finite magnetizations in the presence of an external field.

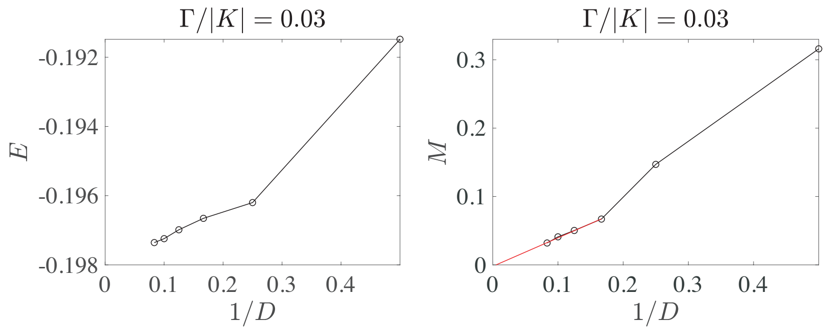

Supplementary FIG. 9: Plots of (left) the variational energy and (right) magnetization as a function of the inverse of the bond dimension D at $\Gamma/|K| = 0.03$ of the K-Γ model, i.e., $(\Gamma', h) = (0, 0)$. The red solid line is the linear fitting curve for the data with $D = 6, 8, 10$ and 12.

Supplementary Note 8: Energy comparison between FM and NP phases

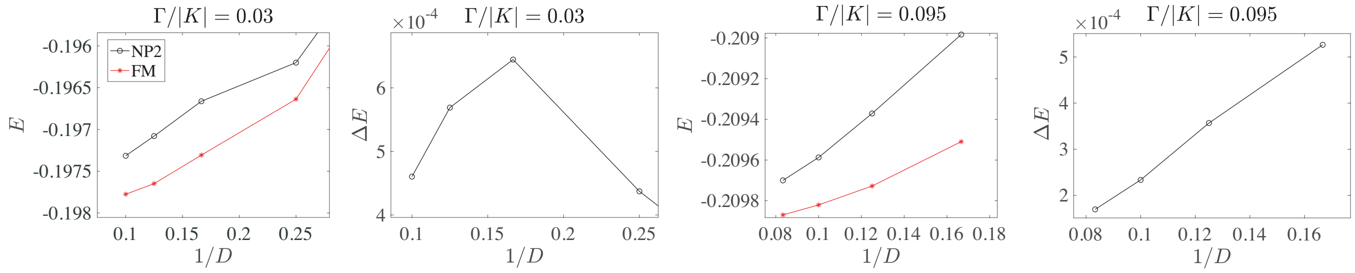

Supplementary FIG. 10: Plots of variational energies E and their differences $\Delta E = E_{\mathrm{NP2}} - E_{\mathrm{FM}}$ of the NP and FM states at $\Gamma/|K| = 0.03$ and 0.095.

As shown and discussed in the main text, both K-Γ and K-Γ-Γ' model exhibit a ferromagnetic phase, where all spins align in the $[1\bar{1}\bar{1}]$-direction, in the zero field limit (see Figs. 1 and 5 in the main text). However, the variational energies between the FM and NP2 states are very close, i.e., the energy difference $\Delta E = E_{\mathrm{NP2}} - E_{\mathrm{FM}} \sim O(10^{-4})$, as shown in the right panel of Supplementary Fig. 10. The results strongly suggest the possibility that the NP2 state may be preferred over the FM states at $h = 0$. This might be captured with much larger bond dimensions.

Supplementary Note 9: Phase transition between the chiral Kitaev spin liquid and the nematic phase in the tilted magnetic field

Without tilting the field ($\theta = 0$), the KSL and the NP phases are clearly distinct each other as shown and discussed in the main text. Since the NP phases are also non-magnetic, one may wonder if those are qualitatively different even with the tilted field where the rotational symmetry is explicitly broken. We have found that those two phases are still distinct each other with the tilted field ($\theta = 5°$). Figure 11 presents the variational energy, the magnetization and the flux expectation value of the optimized states from the initial FM[111], zigzag and SG states at $h = 0.02$ and $\theta = 5°$. The initial FM[111] state flows into the NP2 states well, while the initial SG state keeps the KSL nature in $0.01 \lessapprox \Gamma/|K| \lessapprox 0.07$. As in the case of $\theta = 0$, the results clearly show that there are successive phase transitions between the polarized and zigzag phases: P $\to$ KSL $\to$ NP2 $\to$ Zigzag. In particular, the KSL and NP2 states give certainly distinct magnetizations and flux curves in each phase.

Supplementary Note 10: Coefficients of the area law in the polarized and NP2 phases.

The entanglement entropy in the P and NP2 phases at $h = 0.15$ and $\theta = 5°$ strictly follows the area law, i.e., $S_{\mathrm{vN}} \simeq \alpha L_y$. Here, we show the coefficient α directly in Supplementary Fig. 12. It is almost constant in the P phase while gradually increases with Γ in the NP2 phase.

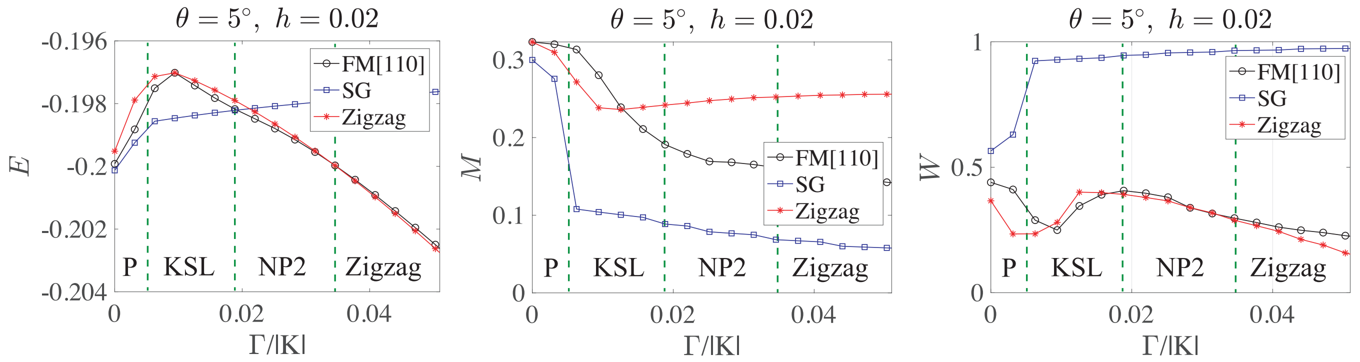

Supplementary FIG. 11: Plots of (left) the variational energy, (center) the magnetization, and (right) the flux expectation value of the optimized states from the initial FM[111], Zigzag and SG states. The green dotted lines stand for the phase boundaries determined by comparing the variational energies. Here, K denotes the chiral KSL phase.

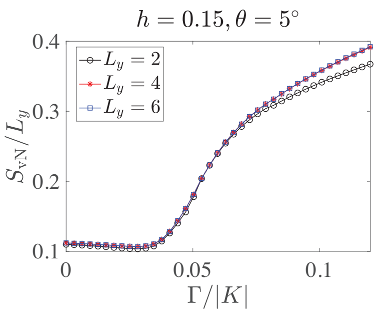

Supplementary FIG. 12: The coefficient of the area law in the entanglement entropy at $h = 0.15$ and $\theta = 0.15°$.

Supplementary References

[1] Hyun-Yong Lee, Ryui Kaneko, Tsuyoshi Okubo, and Naoki Kawashima, "Gapless Kitaev spin liquid to classical string gas through tensor networks," Phys. Rev. Lett. **123**, 087203 (2019).

[2] Li Ern Chern, Ryui Kaneko, Hyun-Yong Lee, and Yong Baek Kim, "Magnetic field induced competing phases in spin-orbital entangled Kitaev magnets," Phys. Rev. Research **2**, 013014 (2020).

[3] Zheng Zhu, Itamar Kimchi, D. N. Sheng, and Liang Fu, "Robust non-abelian spin liquid and a possible intermediate phase in the antiferromagnetic Kitaev model with magnetic field," Phys. Rev. B **97**, 241110 (2018).

[4] Ciarán Hickey and Simon Trebst, "Emergence of a field-driven $U(1)$ spin liquid in the Kitaev honeycomb model," Nature Communications **10**, 530 (2019).

[5] David A. S. Kaib, Stephen M. Winter, and Roser Valentí, "Kitaev honeycomb models in magnetic fields: Dynamical response and dual models," Phys. Rev. B **100**, 144445 (2019).
